# Temperature invariant metasurfaces

**DOI:** 10.1515/nanoph-2023-0075

**Published:** 2023-06-22

**Authors:** Shany Zrihan Cohen, Danveer Singh, Sukanta Nandi, Tomer Lewi

**Affiliations:** Faculty of Engineering, Bar-Ilan University, Ramat-Gan 5290002, Israel; Institute for Nanotechnology and Advanced Materials, Bar-Ilan University, Ramat-Gan 5290002, Israel

**Keywords:** meta-optics, metasurfaces, temperature invariant, thermal dispersion, thermo-optic

## Abstract

Thermal effects are well known to influence the electronic and optical properties of materials through several physical mechanisms and are the basis for various optoelectronic devices. The thermo-optic (TO) effect, the refractive index variation with temperature (d*n*/d*T*), is one of the most common mechanisms used for tunable optical devices, including integrated optical components, metasurfaces, and nano-antennas. However, when a static and fixed operation is required, i.e., temperature invariant performance – this effect becomes a drawback and may lead to undesirable behavior through drifting of the resonance frequency, amplitude, or phase, as the operating temperature varies over time. In this work, we present a systematic approach to mitigate thermally induced optical fluctuations in nanophotonic devices. By using hybrid subwavelength resonators composed from two materials with opposite TO dispersions (d*n*/d*T* < 0 and d*n*/d*T* > 0), we are able to compensate for TO shifts and engineer nanophotonic components with zero effective TO coefficient (d*n*_eff_/d*T* ≈ 0). We demonstrate temperature invariant resonant frequency, amplitude, and phase response in meta-atoms and metasurfaces operating across a wide temperature range and broad spectral band. Our results highlight a path towards temperature invariant nanophotonics, which can provide constant and stable optical response across a wide range of temperatures and be applied to a plethora of optoelectronic devices. Controlling the sign and magnitude of TO dispersion extends the capabilities of light manipulation and adds another layer to the toolbox of optical engineering in nanophotonic systems.

## 1 Introduction

The thermo-optic (TO) coefficient is a macroscopic property defining the refractive index variation with temperature (d*n*/d*T*) and can be assigned to materials, composites, and devices [1, 2]. Many optoelectronic applications utilize this effect as a tuning [3–11], modulating [12–16], or sensing mechanism [17–20], where small changes in temperature lead to resonance wavelength shifts, variations in phase propagation, or other optical properties. Indeed, a plethora of active TO based devices have been successfully implemented in fiber-based devices [21, 22], integrated optics [18, 23] and metasurfaces [3, 4, 6, 7, 24], [25], [26], [27]. While the TO effect offers significant advantages, it may pose a challenge when a consistent and constant optical response is necessary, as the optical characteristics of the device can vary with temperature, resulting in unfavorable performance. In high-*Q* resonant structures such as ring resonators with typical linewidth of ∼10 pm, temperature changes as small as 0.1 K will shift the operating resonance wavelength by 40 pm [23, 28] (i.e., 4 times the resonance linewidth), which would be detrimental to the device operation. Similar effects can also occur in low-*Q* resonant structures which are subjected to large temperature gradients (10 s or 100 s of kelvin degrees), where the optical properties may change dramatically. Extreme temperature gradients inherently exist in space applications [29], however, large and moderate temperature variations may also occur in laser systems [30], thermophotovoltaics, photodetection, and sensing [19, 31]. Temperature variations may be localized – due to, e.g., local heating from intense light sources [5] (due to small absorption arising from e.g., fabrication imperfections), electronic circuits, or non-local, due to changes in ambient temperature, as in the case for space applications or sensors.

**Figure 1:**
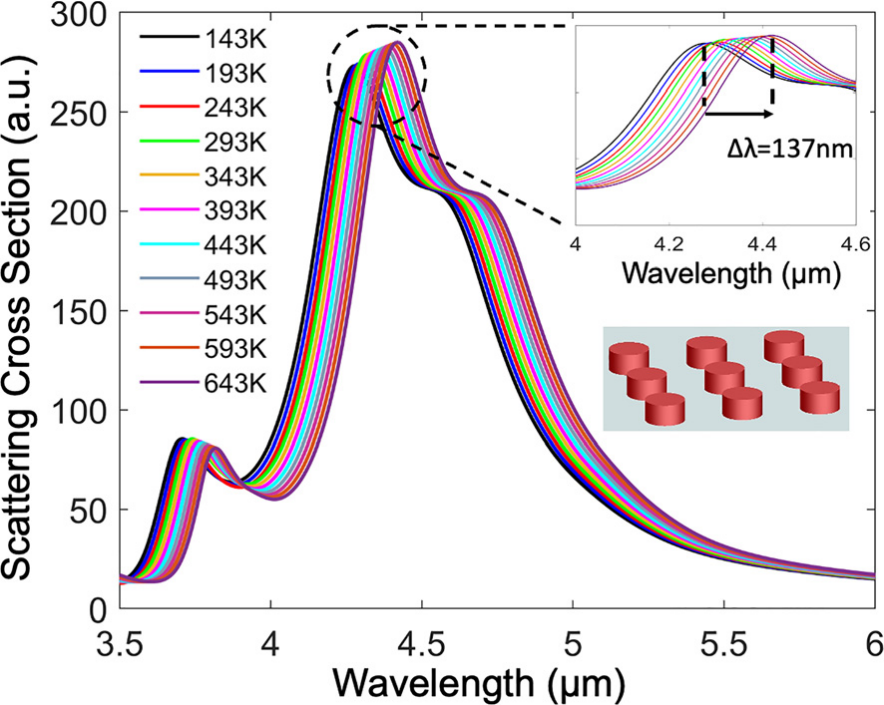
Thermo-optic induced shifts in metasurfaces. A metasurface comprised of silicon disks exhibiting significant resonance red shift (Δ*λ* ≈ 137 nm) with temperature. The unit-cell disk dimensions are *d* (diameter) = 1 µm, *h* (height) = 2.25 µm, the periodicity is Λ = 3.2 μm, and the substrate is BaF_2_.

Consider for example the temperature dependent mid-infrared (MIR) spectra of a silicon disk metasurface shown in Figure 1, plotted for the temperature range 143–643 K. The spectra exhibit strong temperature dependent resonance red shifts (Δ*λ* ≈ 140 nm, 0.3 nm/K) which ultimately result in significant changes in all optical properties (i.e., amplitude, phase). Such strong temperature dependance is a result of the large TO coefficient of silicon (d*n*_Si_/d*T* ∼ 2.5 × 10^−4^/K [1, 6]) and cannot be ignored when the device is subjected to large temperature variations (as shown in Figure 1). The effect of thermo-optically induced shifts becomes increasingly significant for high-*Q* structures, and particularly in ultra-high *Q* resonant structures such as microring, microsphere, and microtoroid resonators. Even small temperature variations in the range of fractions of a kelvin degree can have a detrimental impact on the performance of these devices [32].

Temperature and thermodynamic effects in metasurfaces have been previously studied in space applications [33], laser science [34], thermal emission [33, 35, 36], photodetection, and imaging [37], where eliminating temperature effects in the performance of nanophotonics devices has been only studied in thin films and waveguides [38–40]. However, none of these works provide a solution for subwavelength Mie-resonators or metasurfaces. The work described in [38] deals with engineering temperature independent thermal emission and relies on insulator – metal transition in Mott insulators. Hence it is limited to phase transition materials and does not apply for temperature invariant scattering properties such as amplitude or phase. The work described in [39] deals with spectral design of temperature invariant narrow bandpass filters using 1D thin film geometry for a limited temperature range (20–160 °C), while the work in [38] demonstrates very small TO effect in a waveguiding geometry for a temperature range of 40 °C. Here, we focus on temperature independent meta-optic components that confine light in subwavelength resonators. Furthermore, we also simultaneously engineer temperature invariant resonance frequency, amplitude and phase responses for a wide range of temperatures (up to 500 K) and across a large spectral band.

**Figure 2:**
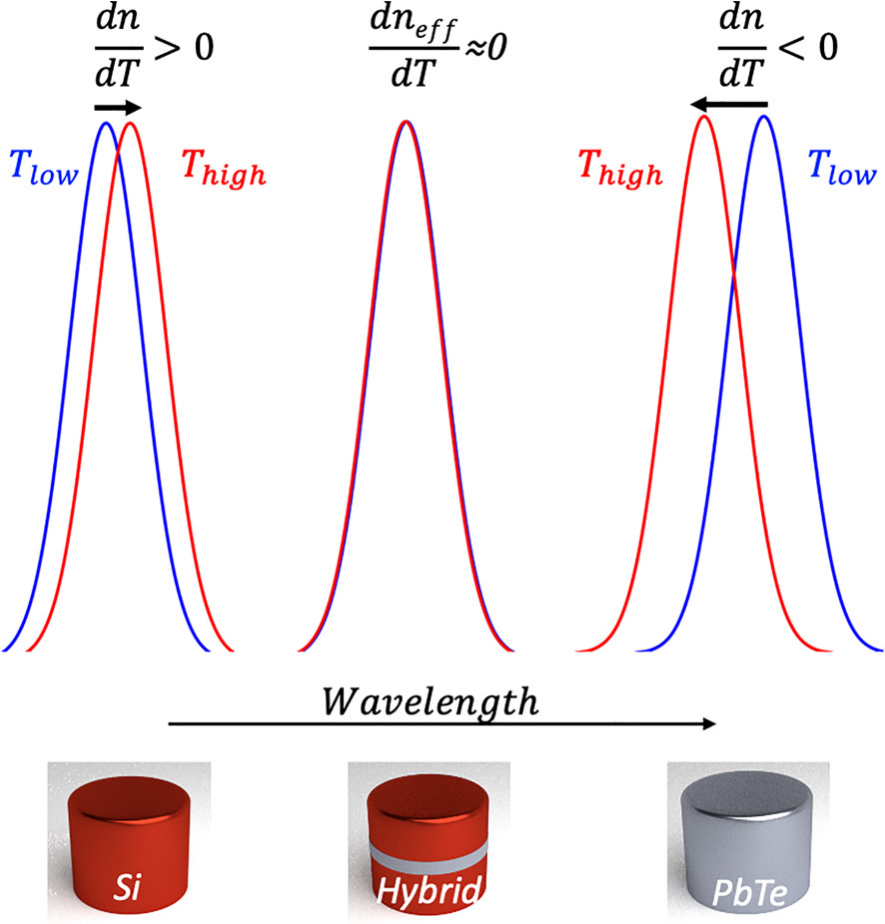
Design concept for temperature invariant nanophotonic components. Hybrid meta-atoms composed from two materials with opposite sign TO coefficients, exhibit temperature invariant response. In this work the positive TO material is Si (d*n*/d*T* > 0) while the negative TO material (d*n*/d*T* < 0) is PbTe. Hybrid Si/PbTe/Si resonators (middle resonator) can be engineered to exhibit temperature invariant performance manifested here by spectral overlap for two extreme temperatures.

Our systematic approach for temperature invariant metasurfaces is based on hybrid dielectric resonators composed from two materials with opposite sign TO coefficients (d*n*/d*T* < 0 and d*n*/d*T* > 0), as illustrated conceptually in Figure 2. Optimizing the resonator geometrical parameters and TO dispersions, we can mutually compensate for TO shifts and engineer meta-atoms and metasurfaces with zero effective TO coefficient (d*n*_eff_/d*T* ≈ 0).

In this work, we focus on all-dielectric structures due to their high efficiency, as they avoid the Ohmic losses that are inherently introduced by metals, or plasmonic materials in general [41]. In principle, metals can be used, however, TO effects in these materials are different. While in semiconductors, the TO effect is mainly determined by variations of the bandgap energy with temperature, the optical constants and TO effects in metals are primarily determined by the properties of their free electrons. Increasing the temperature in metals will alter their complex permittivity which will be manifested in most cases by increased Ohmic losses, resonance broadening, and amplitude damping caused by stronger electron–phonon scattering [37, 42], [43], [44]. Hence, eliminating thermo-optical effects in metal–dielectric or all metallic structures will be challenging, especially for large temperature gradients.

We demonstrate the proposed all-dielectric approach (Figure 2) for a variety of typical nanophotonic components, including Bragg mirrors, single spherical, cubic and disk resonators and finally for large metasurface arrays. Our findings demonstrate that by controlling the sign and magnitude of TO dispersion, it is possible to cancel or mitigate thermally induced shifts in optical systems, leading to increased stability, efficiency, and performance. This approach expands our ability to control light and may broaden the potential applications in nanophotonic systems.

## 2 Results

We divide our results to 1D Bragg mirrors, single Mie resonators of various geometries, and full metasurfaces. For the positive TO material, we selected silicon (Figure 2) – a high index material (*n*_Si_ ≈ 3.42 @5 µm and d*n*_Si_/d*T* = 2.5 [10^−4^/K] at room temperature (RT)) which is transparent across the infrared range [1, 5]. To compensate for the positive TO effect in silicon, the structure must include materials with negative TO coefficients. While most high index dielectrics and semiconductors possess positive TO coefficients, the lead chalcogenide family (PbTe, PbSe, PbS) has both high refractive indices and an anomalous negative TO effect (see Supporting Information Section 1 for more details). We selected PbTe as the negative TO material, as it has the highest refractive index (*n*_PbTe_ = 5.85 @5 µm) and largest TO coefficient (d*n*_PbTe_/d*T* = −13.5 [10^−4^/K] at RT) among the lead chalcogenides [1, 6]. These two materials, silicon and PbTe, form the basic hybrid unit cell in our structures.

In our designs, we take into consideration the chromatic dispersion (*n*(*λ*)) of the refractive indices of both materials, as well as, the temperature and wavelength dispersion of the TO coefficients d*n*/d*T*(*λ*,*T*) [1]. While there are several experimental reports for the temperature dependance of the TO coefficient in Si, spanning hundreds of kelvin degrees [1], this is not the case for PbTe. In fact, the d*n*/d*T* of PbTe was mostly measured around RT [1]. Therefore, we used this reported RT value of d*n*/d*T*(*λ*) in crystalline PbTe [1], for all the range of calculated temperatures. We note that accurate knowledge of the TO coefficients across a large range of temperatures will ultimately determine the performance of temperature invariant devices.

The spectral range of interest in this study lies in the mutual transparent infrared range of both materials (3.8 μm < *λ* < 15.5 μm), away from the fundamental materials bandgaps (*λ*_*g*_ ∼ 1.1 μm for Si and *λ*_*g*_ ∼ 3.8 μm for PbTe, where *λ*_*g*_ is the wavelength corresponding to the energy bandgap). The bandgap wavelength *λ*_*g*_ ∼ 3.8 μm of PbTe is the lower limit where it can be considered transparent. For shorter wavelengths, PbTe will start absorbing and its refractive index will become complex, resulting in resonance broadening and amplitude damping, which will ultimately affect the ability to compensate for the TO effects in silicon. The long wavelength limit is determined by multiphonon absorption in Si, typically around ∼15.5 μm [45], leading to the transparency spectral range 3.8 μm < *λ* < 15.5 μm of the hybrid structures presented here.

### 2.1 1D structures: Bragg mirrors

Bragg mirrors are widely used in various applications due to their high reflectivity and low losses [39, 46]. Standard Bragg mirrors incorporate stacks of alternating layers of high and low refractive indices and can be designed for a specific central wavelength as well as to function as bandpass or bandstop filters. Here, we implement the same approach, however, the layers alternate not only in the refractive index, but also in the sign of the TO coefficient.

**Figure 3:**
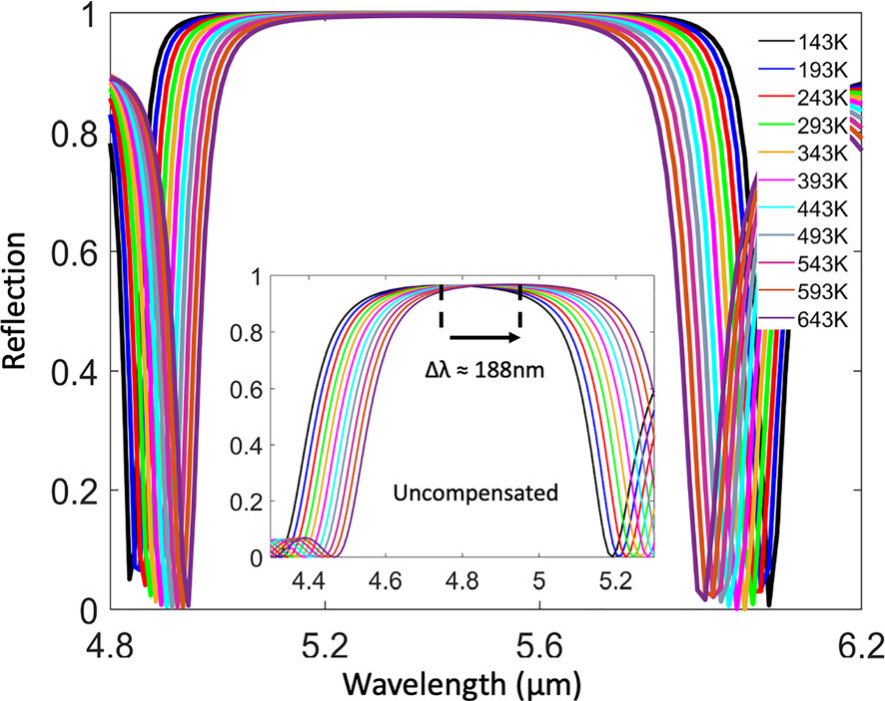
Temperature independent Bragg mirror incorporating 11 alternating layers of PbTe–Si with thicknesses *d*_PbTe_ = 0.19 µm, *d*_Si_ = 1.25 µm. The central resonance wavelength *λ* = 5.36 µm exhibits high reflectivity as well as temperature invariance behavior. Inset shows a similar Bragg mirror, comprised of 11 alternating layers of SiO_2_–Si (having the exact same thicknesses *d*_SiO2_ = 0.19 µm, *d*_Si_ = 1.25 µm), which is uncompensated for TO shifts, exhibiting resonance wavelength shifts of Δ*λ* ≈ 188 nm.

The infrared spectra of an 11-layer stack of PbTe/Si are presented in Figure 3. While all resonances across the broad 4–12 µm spectrum exhibit very low temperature dependence, the results were optimized for the central wavelength *λ*_0_ = 5.36 µm. Indeed, at this resonance wavelength, the structure demonstrates extremely high reflectivity (*R* ≈ 0.99) for all the simulated temperatures, throughout a wide bandpass of Δ*λ* ≈ 880 nm, as well as temperature invariant performance across a 500 K temperature span, with no spectral shift of the central resonance wavelength. For comparison, the inset of Figure 3 shows the spectra of a similar infrared SiO_2_/Si uncompensated Bragg reflector, exhibiting TO resonance shifts of Δ*λ* ≈ 188 nm.

Interestingly, although the central resonance wavelength of the PbTe/Si Bragg reflector is fixed (across all temperatures), we observe bandpass narrowing of Δ*λ*_max_ ≈ 125 nm as temperature increases. This bandpass narrowing is a result of the reduction in refractive index contrast with temperature; namely, n_PbTe_ decreases with temperature, while n_Si_ increases.

### 2.2 2D structures: three-layer hybrid Mie resonators

The power in manipulating free space light lies in two-dimensional metastructures. Mie resonator meta-atoms form the basic unit cell building blocks for metasurfaces and metamaterials [47] and are also excellent scatterers as single or ensembles of nano-antennas [48]. We start by designing temperature invariant three-layer hybrid single Mie-resonators spanning various geometries. We select three common geometries – sphere, disk, and cubic resonators, and carefully engineer each of the structures to eliminate the TO dispersion (d*n*_eff_/d*T* ≈ 0) for a given mode. The structures are symmetric in the *x*–*y* plane, and the incident beam is *x*-polarized propagating along the *z*-axis. The unit cell in all structures is composed of three Si/PbTe/Si layers and the scattering spectra were obtained using finite difference time domain (FDTD) solver (see Supporting Information Section 4). It should be noted that such three-layer Mie-resonator structures can be fabricated using standard top–down or bottom–up fabrication techniques [49–52].

#### 2.2.1 Spherical multilayer core–shell hybrid Mie-resonators

**Figure 4:**
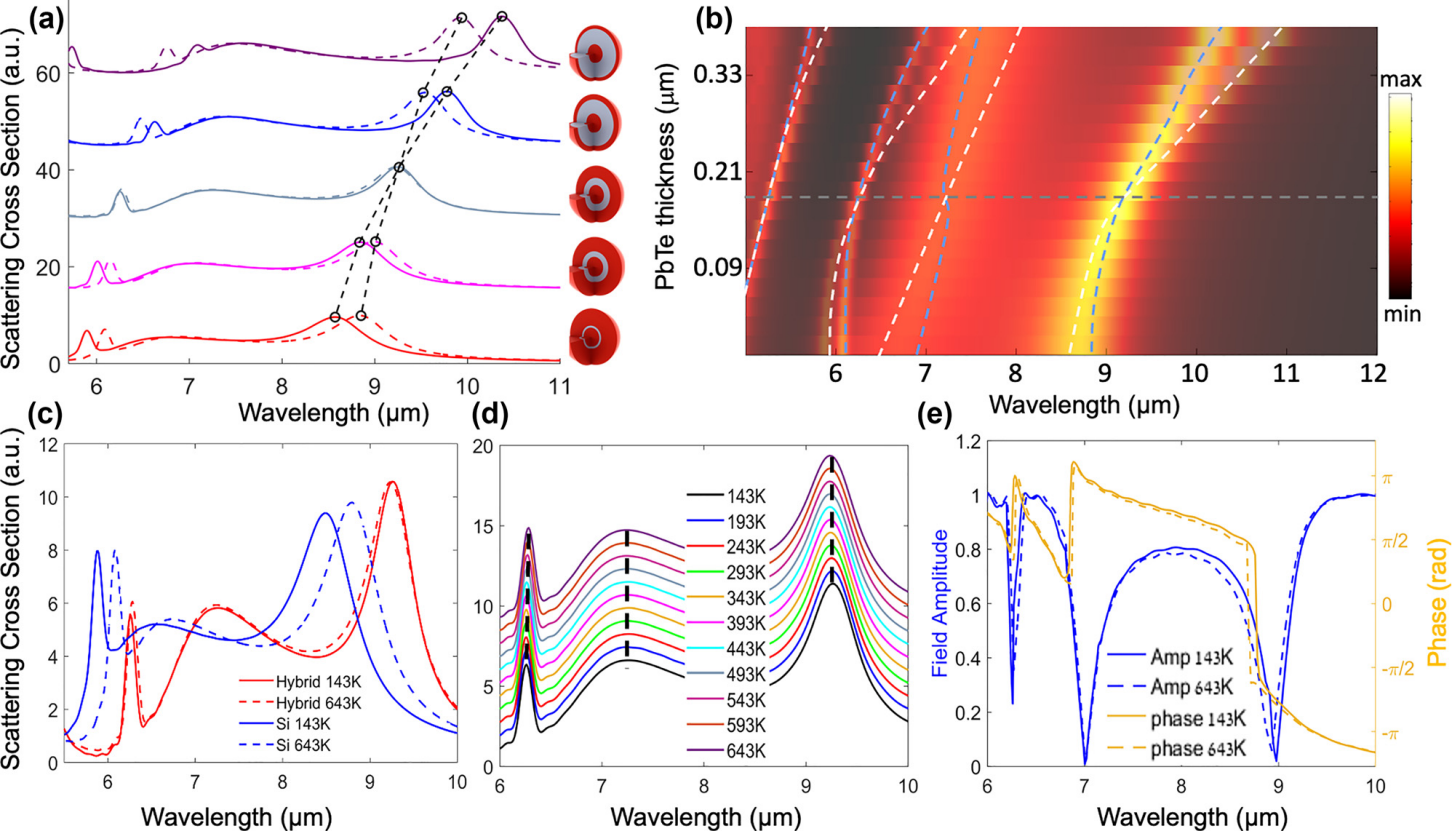
Design and properties of temperature invariant multilayer Si/PbTe/Si spherical resonators. (a) Optimization of the Si/PbTe/Si structure by plotting the spectra for the two extreme temperatures *T*_L_ = 143 K (solid lines) and *T*_H_ = 643 K (dashed lines), varying the PbTe shell thickness (spectra are vertically shifted for visibility), while keeping the inner (core) Si diameter (*d*_Si,1_ = 0.86 µm) and the overall diameter of the structure fixed (*d*_tot_ = 2.4 µm). The dashed black lines follow the spectral position of the fundamental MD mode. Optimum thickness is reached when the two spectra completely overlap, resulting in temperature invariant response (b) quantitative color map of the same optimization process. The spectra for the two extreme temperatures are plotted together, while varying the PbTe thickness. The color represents the scattering intensity at each point in the map. The dashed white and light blue lines follow the spectral evolution of the first four Mie-resonant modes for the low temperature *T*_L_ = 143 K (white), and high temperature *T*_H_ = 643 K (light blue), for varying PbTe shell thickness. The horizontal dashed line corresponds to the crossing point of the two branches (*T*_L_ and *T*_H_), representing the optimized PbTe thickness for temperature invariant response. (c) Comparison between the scattering spectra of the optimized hybrid structure (red) and the spectra of an uncompensated Si sphere (blue) of the same size, for the two extreme temperatures (*T*_L_ = 143 K and *T*_H_ = 643 K). For the optimized hybrid spherical resonator the inner Si radius is *r*_Si_ = 0.43 µm, the PbTe radius is *r*_PbTe_ = 0.62 µm and the outer Si layer radius is *r*_Si_ = 1.2 µm. (d) Scattering spectra of the optimized multilayer spherical resonator, demonstrating fixed spectral position across a Δ*T* = 500 K temperature swing for increments of 50 K (the spectra are vertically shifted along the *y*-axis for visibility). (e) Transmission amplitude and phase spectra at *T* = 143 K and *T* = 643 K, respectively, calculated for the hybrid unit-cell using periodic boundary conditions along *x*–*y* plane (see Supporting information for more details). Negligible variations in both amplitude and phase are observed, demonstrating that in addition to resonant frequency, both the phase and the amplitude remain the same between these the two extreme temperatures (143 K vs. 643 K).

The spectra of multilayer core–shell Si/PbTe/Si spherical Mie resonators are presented in Figure 4(a) and illustrate the design and optimization of the structure for obtaining temperature invariant response (the layered structures are shown in the inset, red colors represent the Si core and outer shell, gray is the PbTe inner shell). In this example, it was done through sweeping the inner PbTe shell thickness, while keeping the inner (core) Si diameter (*d*_Si,1_ = 0.86 µm) and the overall diameter of the structure fixed (*d*_tot_ = 2.4 µm).

Figure 4(a) presents the scattering spectra for the two extreme temperatures *T*_L_ = 143 K (solid lines) and *T*_H_ = 643 K (dashed lines), varying the PbTe shell thickness (the spectra are shifted vertically for visibility). The goal of the optimization process is to increase the thickness of the PbTe layer in the compound, until the spectra at the two extreme temperatures *T*_L_ = 143 K (solid lines) and *T*_H_ = 643 K, will completely overlap. The increase in the PbTe shell thickness results in two effects. First, the total effective index of the structure increases (due to the larger fraction of the high index PbTe in the compound), resulting in a red shift of the spectra for both temperatures. This explains the spectral red shift for both temperatures (solid lines and dashed lines) as we move up in the plot for structures with larger PbTe volume. Second, as the PbTe thickness increases, the effective TO coefficient (d*n*_eff_/d*T*) in the multilayer structure decreases, reducing the relative shift between the spectra of the two extreme temperatures. The optimum thickness is reached when the two spectra completely overlap, resulting in temperature invariant response. Further increasing the thickness of the PbTe beyond the optimum point, results in splitting of the two spectra once more. Figure 4(b) presents a quantitative color map of this same process, where we plot together the spectra for the two extreme temperatures (*T*_L_ = 143 K and *T*_H_ = 643 K), for varying PbTe thicknesses (the color represents the scattering intensity). The dashed lines follow the spectral mode evolution of the first four Mie-resonant modes, for *T*_L_ = 143 K (white) and *T*_H_ = 643 K (light blue), for varying PbTe shell thicknesses. The horizontal gray dashed line corresponds to the crossing point of the two branches (*T*_L_ and *T*_H_) of each Mie mode, representing the optimized PbTe thickness for temperature invariant response.

**Figure 5:**
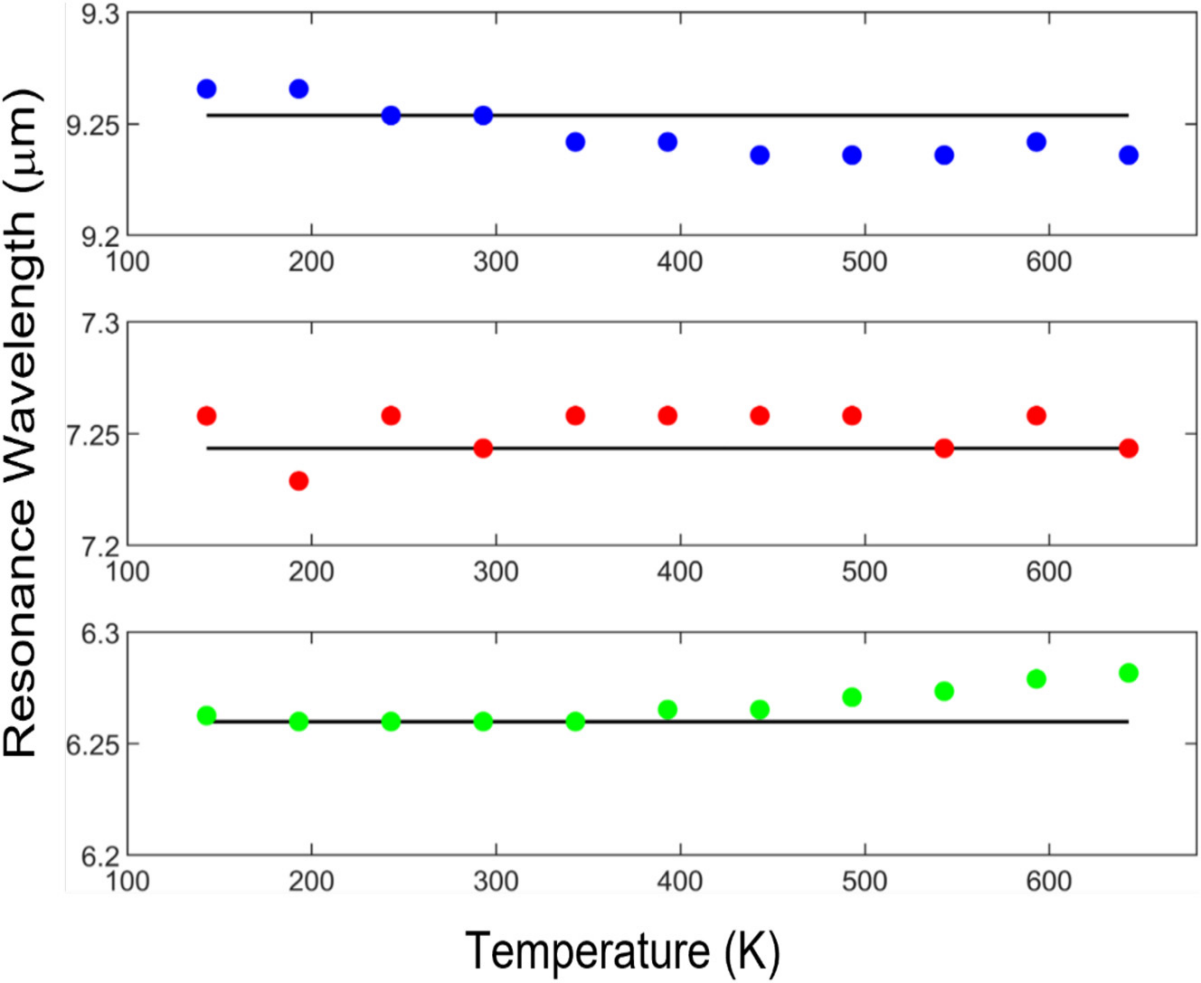
Extracted resonance wavelength versus temperature for MD (blue), ED (red) and MQ (green) resonances in spherical resonators shown in Figure 4(d). Temperature independent response is manifested by a small FOM of all resonances (*S*_max_ < 0.003).

Figure 4(c) compares the scattering spectra of the optimized hybrid structure, to the spectra of a Si sphere of the same diameter, for the two extreme temperatures (*T*_L_ = 143 K and *T*_H_ = 643 K). While the Si sphere exhibits TO shift of ∼300 nm for the Δ*T* = 500 K temperature difference, the hybrid sphere presents overlapping spectra with temperature independent resonance wavelength positions. Figure 4(d) presents the optimized hybrid sphere spectra varying the temperature in increments of 50 K (the spectra are vertically shifted along the *y*-axis for visibility). It is clear that the optimization procedure resulted in temperature invariant response for all the temperatures within the studied range. Namely, the spectral position of the first three resonant modes: the magnetic dipole (MD), electric dipole (ED), and magnetic quadrupole (MQ), are fixed. In addition to the spectral response, both the phase and the amplitude remain the same, as presented in Figure 4(e). This is exemplified by the overlapping amplitude and phase response across the 6–10 µm spectral range, at 143 K and 643 K, respectively. To quantify the resonance wavelength shifts, we define a normalized figure of merit (FOM) *S*_max_ = Δ*λ*/*λ*_0_ where *λ*_0_ is the resonance wavelength at RT, and Δ*λ* is the maximum resonance shift compared to the resonance wavelength at RT. Figure 5 present the extracted temperature dependent resonance wavelengths of the MD, ED, and MQ modes of Figure 4(d).

Evidently, temperature independent response is manifested by a small FOM of all resonances (|*S*_max_| < 0.003) for this spherical resonator. Complete cancelation of the effective TO effect in the resonator (d*n*_eff_/d*T* = 0) is limited to ∼Δ*T* = 150 K. For example, the MQ mode (green dots, lower panel of Figure 5), was optimized to exhibit zero resonance wavelength shift d*λ*/d*T* = 0 for 193 K < *T* < 343 K. For comparison, with no TO correction, a similar sized silicon resonator would have exhibited a Δ*λ* = 75 nm resonance wavelength shift for the same Δ*T* = 150 K. Furthermore, analysis of the temperature dependent electric and magnetic field distributions for all the resonances reveals that the resonant mode field profiles also remain fixed across the full 500 K temperature gradients (see Supporting Information Section 2.1).

#### 2.2.2 Hybrid disk and cubic Mie-resonators

**Figure 6:**
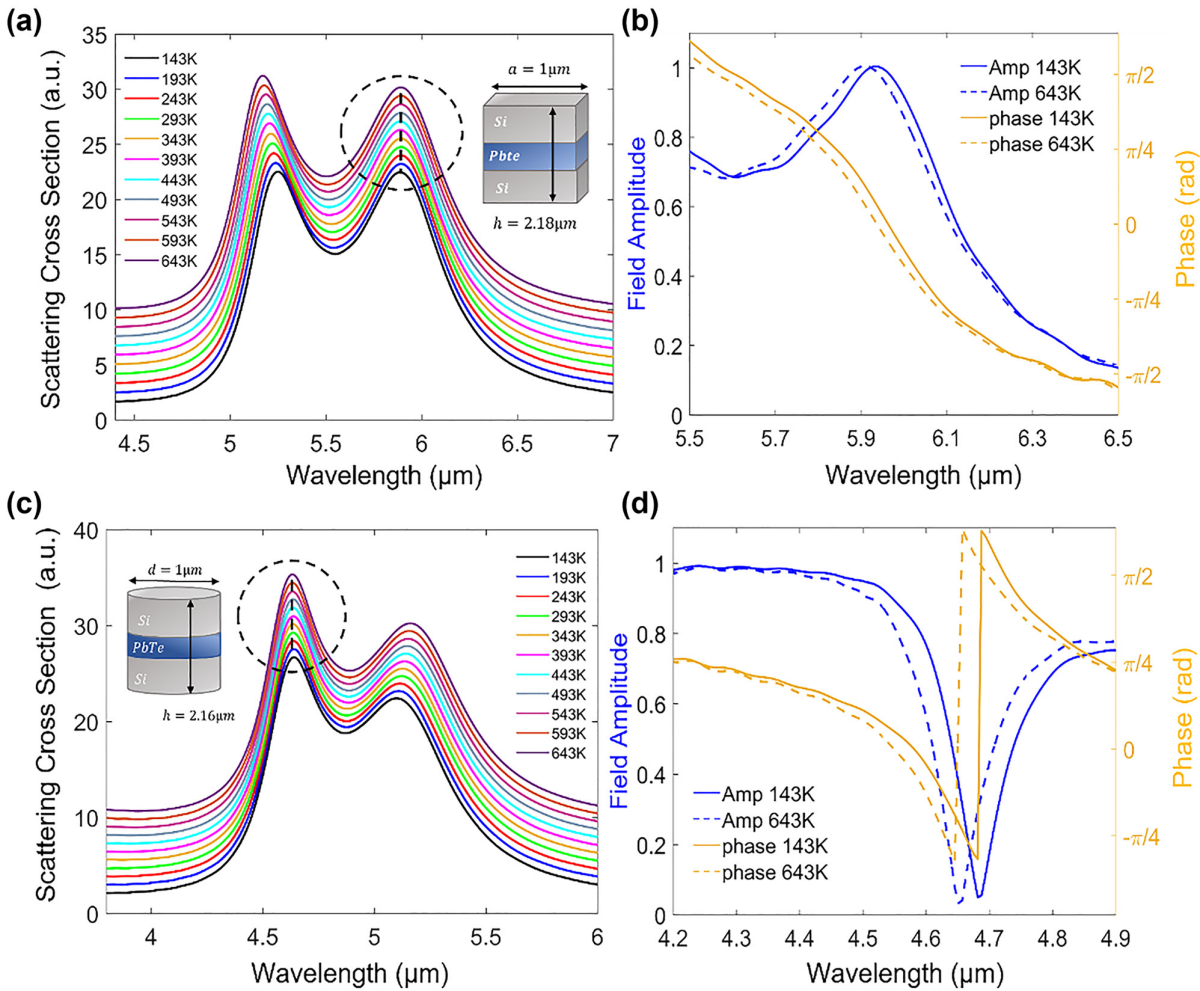
Disk and cubic multilayer hybrid resonators. (a) Scattering spectra of a cubic resonator demonstrating pinned resonance wavelength behavior across the 143 K–643 K temperature range. The spectra are vertically shifted along the *y*-axis for visibility. The hybrid cubic resonator dimensions are *h*_Si,1_ = 0.92 µm, *h*_PbTe_ = 0.34 µm and *h*_Si,2_ = 0.92 µm, while the side is *a* = 1 µm. The spectra demonstrate temperature independent behavior for the first mode (dashed circle). (b) Reflection amplitude and phase for *T* = 143 K (blue) and *T* = 643 K (orange), exhibiting minor variations in both the phase and amplitude for the two extreme temperatures. (c) Hybrid disk scattering spectra, demonstrating temperature independent behavior for the second mode (dashed circle). The spectra are vertically shifted along the *y*-axis for visibility. The disk dimensions are *h*_Si,1_ = 0.96 µm, *h*_PbTe_ = 0.24 µm and *h*_Si,2_ = 0.96 µm, while the radius is *r* = 500 nm. (d) Transmission amplitude and phase around the second (ED) mode. Minor variations in both amplitude and phase are visible.

Disk and cubic resonators are the most common unit-cells in meta-optics and possess additional degrees of freedom compared to spheres, as their geometry breaks the spherical symmetry. The FDTD scattering spectra, along with the amplitude and phase response of cubic and disk hybrid Mie-resonators are shown in Figure 6. The breaking of spherical symmetry, especially along the *z* axis compared to the *x*–*y* plane, is known to change the dispersion of different Mie resonant modes and is often used to design unidirectional Huygens metasurfaces [53, 54]. Here, the different polarization of Mie-resonances leads to different temperature dispersion of resonances which makes it more challenging to simultaneously design temperature invariant response for two or more resonant modes. Hence, we focus on designing temperature invariant response around one resonance mode per structure, where canceling the TO dispersion in each structure is optimized for one dipolar mode.

The spectra presented in Figure 6(a) and (c), demonstrate minor wavelength variations over large temperature span (Δ*T* = 500 K), for a particular dipolar mode of the individual resonator. For comparison, uniform Si structures of identical sizes (see Supporting information Figure S5), exhibit significant temperature sensitivity leading to resonance wavelength shifts of up to 190 nm. *S*_max_ for the fundamental mode in the cubic structure (circled in Figure 5(a)), receives a value of |*S*_max_| = 0.0012 while |*S*_max_| = 0.0013 is obtained for the 2nd mode in the disk geometry (highlighted by a circle in Figure 6(c)). The field profiles for these structures were also calculated and can be found in the Supporting Information Section 2.2. Furthermore, both structures show minor variations in phase and amplitude (Figure 6(b)–(d)), between the two extreme temperatures (*T* = 143 K and *T* = 643 K). These variations are mostly observed around the abrupt changes at the resonance wavelengths and are slightly larger for the disk geometry.

**Figure 7:**
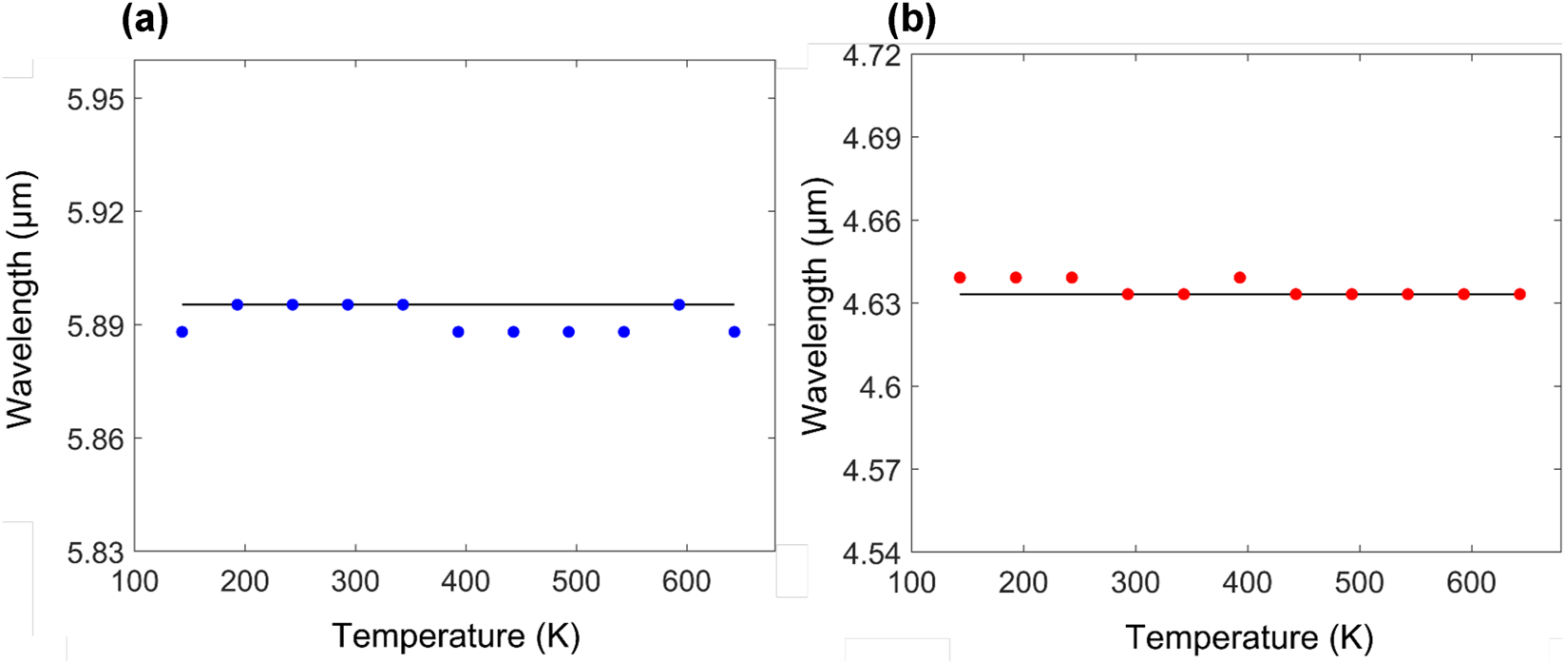
Extracted resonance wavelength versus temperature for (a) fundamental mode in the cubic resonator shown in Figure 6(a) and (b) the 2nd mode of the disk resonator shown in Figure 6(c). Temperature independent response is manifested by near zero values of *S*_max_ (*S*_max_ ≈ 0.0013).

Figure 7 summarizes the temperature dependent resonant wavelength properties of cubic and disk hybrid Mie resonators. Temperature independent response is manifested by near zero values of *S*_max_ for both structures, showcasing the ability to tailor the unit cell parameters in order to mitigate temperature effects per given mode. Similar to the spherical resonator, complete elimination of the effective thermo-optic response can be achieved for a reduced temperature range of Δ*T* ≈ 200 K. This can be seen in Figure 7b, where the disk resonator exhibits zero resonance wavelength shifts (d*λ*/d*T* = 0), between *T* = 443 K and *T* = 643 K. Similarly, the parameter sweep process can be further optimized for any of light’s degrees of freedom (e.g., phase, amplitude, polarization, angular momentum, etc.).

#### 2.2.3 Hybrid disk metasurfaces

Following the study of temperature invariant response in single meta-atoms, we move forward to implementing full metasurface arrays. Such meta-optic components could be integrated into nanophotonic and electro-optic devices (filters, beam shaper, lenses, etc.) providing stable, robust, and temperature independent response.

**Figure 8:**
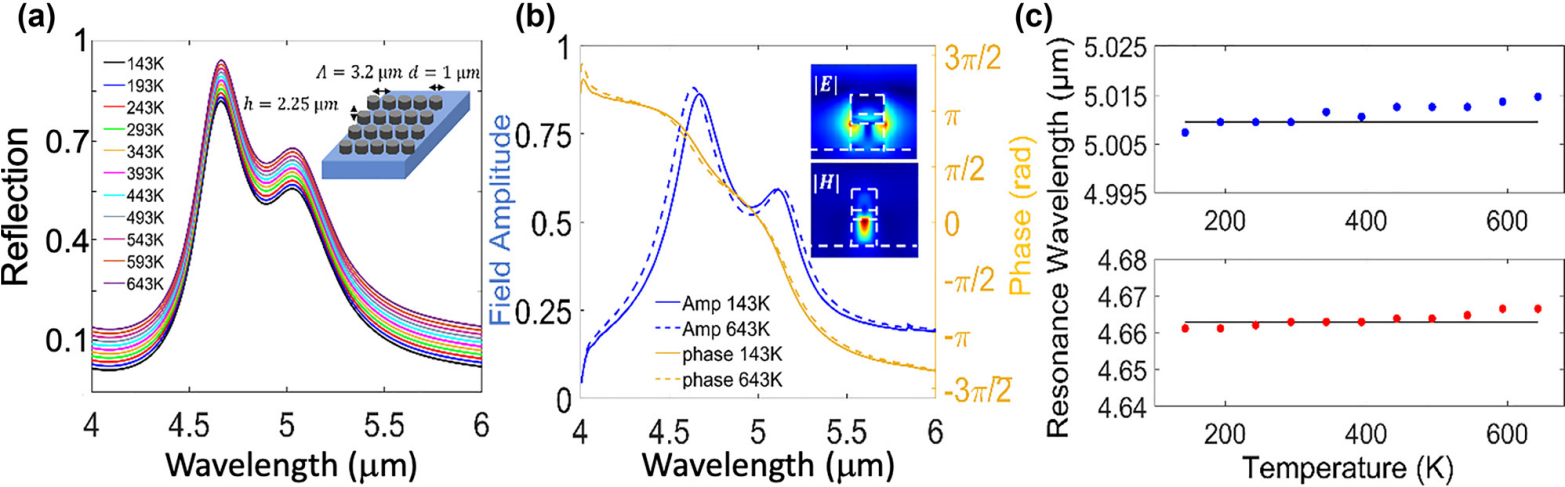
Temperature invariant metasurfaces. (a) Hybrid disk metasurface reflection spectra demonstrating near perfect temperature invariant behavior (The spectra are slightly shifted vertically along the *y*-axis for visibility). The metasurface geometry is illustrated in the inset. The thicknesses of the Si/PbTe/Si layers in unit-cell are *h*_Si,1_ = 1 µm, *h*_PbTe_ = 0.25 µm, and *h*_Si,2_ = 1 µm, the diameter *d* = 1 µm and the periodicity in the array is Λ = 3.2 μm. (b) Hybrid disk metasurface reflection spectrum, demonstrating pinned behavior of the amplitude and phase of *T* = 143 K versus *T* = 643 K. Field profiles of the fundamental mode are depicted in the inset. (c) Extracted resonance wavelength versus temperature for the fundamental (blue, upper panel) and the 2nd (red, lower panel) modes of the hybrid disk metasurface. Temperature independent response is manifested by near zero values of the FOM (*S*_max_ ≤ 0.001).

Figure 8(a) presents FDTD calculated spectra for a metasurface comprised of hybrid disk resonator unit cells. BaF_2_ was chosen as the substrate material, due to its low thermo-optic response (d*n*/d*T* ≈ −19 × 10^−6^[1/K]) [55], low refractive index, as well as a wide transparency window in the visible to MIR range [56]. The metasurface layout along with the lattice constant (Λ = 3.2 µm) are shown in the inset of Figure 8(a). Since the periodicity of the structure is smaller than the free space wavelength of the incident light, the overall scattering properties are mostly inherited by the single unit cell resonator [53, 57]. For very small lattice constants, the inter-particle interactions would be more significant, however these conditions were not considered here.

Our results demonstrate that the scattering cross section, amplitude, resonance wavelength, as well as the phase, for the two fundamental modes – all maintain their RT values (Figure 8(a) and (b)). Minor resonance wavelength shifts of Δ*λ*_max_ < 5 nm are observed for the two resonant modes, as can be seen in Figure 8(c). The corresponding *S*_max_ values for the two fundamental modes are *S*_max_ = 0.001 and *S*_max_ = 7.72 × 10^−4^, for 143 K < *T* < 643 K, respectively. Perfect temperature invariant performance (Δ*λ* = 0, *S*_max_ = 0) is observed between *T* = 293 K and *T* = 393 K (lower panel in Figure 8(c)), while maximum resonance shift as small as Δ*λ* = 0.9 nm, is obtained for a wider temperature range Δ*T* = 250 K (243 K < *T* < 493 K). A similar uniform Si metasurface with no TO correction (see Figure 1(a) for comparison), would have exhibited a Δ*λ* ≈ 75 nm for the same temperature gradient Δ*T* = 250 K.

Figure 8(b) presents the reflection phase, demonstrating temperature invariant full 2*π* phase coverage across the two fundamental modes. Electric and magnetic field profiles of the fundamental dipole mode are presented in the inset of Figure 8(b), exhibiting the typical field distribution of MD, similar to the field patterns of single disk resonators (see Supporting Information Section 2.2). Spanning 2*π* phase coverage is fundamental to meta-optic design as it allows implementing a plethora of optical functionalities. The capability to maintain temperature independent resonant wavelength along with 2*π* phase coverage in a realistic meta-optic device, showcases the strength of the proposed design and demonstrates the potential to implement TO dispersion engineering in nanophotonics devices.

## 3 Conclusions

In this work we propose a method to eliminate thermally induced shifts in the optical properties of nanophotonic and meta-optic devices. Our approach is based on compound meta-atom unit cells composed from at least two materials with opposite TO coefficients, allowing to engineer near zero effective TO coefficient (d*n*_eff_/d*T* ≈ 0). Following the optimization of the hybrid unit-cell, it is possible to cover any spectral band in the transparency window of the materials (3.8 μm < *λ* < 15.5 μm), by scaling the total size of the unit (while keeping the same volume ratio of the layers). We demonstrate temperature invariant response for multilayer Bragg mirrors and single meta-atoms of various geometries. We also demonstrate very small variations in the resonance frequency (*S*_max_ < 0.001), amplitude and phase of these resonators, across large temperature gradients spanning 500 K degrees. Thermally induced optical shifts are at least an order of magnitude smaller than the shifts of similar resonators with no TO shift correction.

The temperature invariant capabilities of full metasurface arrays surpass single resonators, due to the reduction in the scattering channels and increase in resonance quality factors. Hybrid disk metasurfaces exhibited near perfect temperature invariant response with resonance wavelength sensitivity of d*λ*_eff_/d*T* ≈ 5 pm/K, for the temperature range 143 K < *T* < 643 K. Peak performance for this metasurface was achieved at *S*_max_ = 7.72 × 10^−4^, which is also the best value reported here for any of the of the studied nanophotonic structures. For relaxed temperature gradient conditions of Δ*T* ≈ 150 K, we achieved perfect zero effective thermo-optic response with *S*_max_ ≈ 0, completely eliminating TO effects. It should be noted that due to the in-plane symmetry in our structures and the normal incident beam, the output response is polarization insensitive. However, for asymmetric resonators, unit-cell arrangements that break the in-plane symmetry, or more complex excitation conditions—TO compensation will depend on the incident beam polarization. In these cases, polarization insensitive response will require more complex unit cells that provide extra degrees of freedom.

In summary, our approach for temperature invariant engineering in single meta-atoms and meta-optics devices provides additional degrees of freedom to optical design capabilities, allowing to compensate for and optimize TO dispersions effecting resonance wavelength, amplitude and phase. We demonstrate that near perfect temperature invariant performance can be achieved for large temperature variations spanning 500 K degrees, which would have otherwise significantly altered the optical properties of the device. These results may pave the way for temperature invariant response in a vast array of applications which are sensitive to temperature variations, enhancing efficiency, stability, and performance.

## Supplementary Material

Supplementary Material Details

## References

[1] Ghosh G. (1998). *Handbook of Thermo-Optic Coefficients of Optical Materials and Applications*.

[2] Zograf G. P., Petrov M. I., Makarov S. V., Kivshar Y. S. (2021). All dielectric thermonanophotonics. *Adv. Opt. Photon*.

[3] Archetti A., Lin R.-J., Restori N., Kiani F., Tsoulos T. V., Tagliabue G. (2022). Thermally reconfigurable metalens. *Nanophotonics*.

[4] Assadillayev A., Hinamoto T., Fujii M., Sugimoto H., Raza S. (2021). Thermal near-field tuning of silicon Mie nanoparticles. *Nanophotonics*.

[5] Tsoulos T. v., Tagliabue G. (2020). Self-induced thermo-optical effects in silicon and germanium dielectric nanoresonators. *Nanophotonics*.

[6] Lewi T., Evans H. A., Butakov N. A., Schuller J. A. (2017). Ultrawide thermo-optic tuning of PbTe meta-atoms. *Nano Lett*..

[7] Lewi T., Butakov N. A., Schuller J. A. (2018). Thermal tuning capabilities of semiconductor metasurface resonators. *Nanophotonics*.

[8] Armani D., Min B., Martin A., Vahala K. J. (2004). Electrical thermo-optic tuning of ultrahigh-Q microtoroid resonators. *Appl. Phys. Lett*..

[9] Montesinos-Ballester M., Liu Q., Vakarin V. (2019). On-chip fourier-transform spectrometer based on spatial heterodyning tuned by thermo-optic effect. *Sci. Rep.*.

[10] Pruessner M. W., Stievater T. H., Ferraro M. S., Rabinovich W. S. (2007). Thermo-optic tuning and switching in SOI waveguide Fabry-Perot microcavities. *Opt. Express*.

[11] Zangeneh Kamali K., Xu L., Gagrani N. (2023). Electrically programmable solid-state metasurfaces via flash localised heating. *Light Sci. Appl.*.

[12] Sun J., Timurdogan E., Yaacobi A., Hosseini E. S., Watts M. R. (2013). Large-scale nanophotonic phased array. *Nature*.

[13] Yu H., Qiu F. (2022). Compact thermo-optic modulator based on a titanium dioxide micro-ring resonator. *Opt. Lett*..

[14] Lee B. S., Zhang M., Barbosa F. A. S. (2017). On-chip thermo-optic tuning of suspended microresonators. *Opt. Express*.

[15] Cao Y., Sahin E., Choi J. W. (2021). Thermo-optically tunable spectral broadening in a nonlinear ultra-silicon-rich nitride Bragg grating. *Photonics Res.*.

[16] Chung S., Nakai M., Hashemi H. (2019). Low-power thermo-optic silicon modulator for large-scale photonic integrated systems. *Opt. Express*.

[17] Ge Y., Liu Q., Chang J., Zhang J. (2013). Optical fiber sensor for temperature measurement based on silicon thermo-optics effect. *Optik*.

[18] Tang T., Luo L. (2016). Refractive index sensor of mach–zehnder interferometer based on thermo-optic effect of SOI waveguide. *Optik*.

[19] Wang M., Perez-Morelo D. J., Aksyuk V. (2021). Overcoming thermo-optical dynamics in broadband nanophotonic sensing. *Microsyst. Nanoeng.*.

[20] Lee S.-M., Ahn K.-C., Sirkis J. S. (2001). Planar optical waveguide temperature sensor based on etched Bragg gratings considering nonlinear thermo-optic effect. *J. Mech. Sci. Technol.*.

[21] Anand V. R., Mathew S., Samuel B., Radhakrishnan P., Kailasnath M. (2017). Thermo-optic tuning of whispering gallery mode lasing from a dye-doped hollow polymer optical fiber. *Opt. Lett*..

[22] Sun B., Zhao J., Wang Y. (2015). Broadband thermo-optic switching effect based on liquid crystal infiltrated photonic crystal fibers. *IEEE Photonics J.*.

[23] Liu X., Ying P., Zhong X. (2020). Highly efficient thermo-optic tunable micro-ring resonator based on an LNOI platform. *Opt. Lett*..

[24] Afridi A., Canet-Ferrer J., Philippet L., Osmond J., Berto P., Quidant R. (2018). Electrically driven varifocal silicon metalens. *ACS Photonics*.

[25] Celebrano M., Rocco D., Gandolfi M. (2021). Optical tuning of dielectric nanoantennas for thermo-optically reconfigurable nonlinear metasurfaces. *Opt. Lett*..

[26] Rahmani M., Xu L., Miroshnichenko A. E. (2017). Reversible thermal tuning of all-dielectric metasurfaces. *Adv. Funct. Mater.*.

[27] Rocco D., Gandolfi M., Tognazzi A. (2021). Opto-thermally controlled beam steering in nonlinear all-dielectric metastructures. *Opt. Express*.

[28] Zhang Y., Hu X., Chen D. (2018). Design and demonstration of ultra-high-Q silicon microring resonator based on a multi-mode ridge waveguide. *Opt. Lett*..

[29] Kaushal H., Kaddoum G. (2017). Optical communication in space: challenges and mitigation techniques. *IEEE Commun. Surv. Tutorials*.

[30] Cheung N. H. (1991). Pulsed laser heating of thin films: an efficient algorithm for computing temperature profiles. *J. Appl. Phys*..

[31] Cocorullo G., della Carte F. G., Rendina I., Sarro P. M. (1998). A thermo-optic effect exploitation in silicon microstructures. *Sens. Actuator A Phys*.

[32] Campione S., Liu S., Basilio L. I. (2016). Broken symmetry dielectric resonators for high quality factor fano metasurfaces. *ACS Photonics*.

[33] Feng D., Jaffe G. R., Yin S. (2022). Characterization of temperature-dependent optical properties of materials for metasurfaces in extreme conditions. *Proc. SPIE*.

[34] Holdman G. R., Jaffe G. R., Feng D., Jang M. S., Kats M. A., Brar V. W. (2022). Thermal runaway of silicon-based laser sails. *Adv. Opt. Mater.*.

[35] Howes A., Nolen J. R., Caldwell J. D., Valentine J. (2020). Near-unity and narrowband thermal emissivity in balanced dielectric metasurfaces. *Adv. Opt. Mater.*.

[36] Park J., Kang J.-H., Liu X. (2018). Dynamic thermal emission control with InAs-based plasmonic metasurfaces. *Sci. Adv.*.

[37] Tang Y.-L., Yen T.-H., Nishida K. (2021). Mie-enhanced photothermal/thermo-optical nonlinearity and applications on all-optical switch and super-resolution imaging [invited]. *Opt. Mater. Express*.

[38] King J. L., Shahsafi A., Zhang Z. (2022). Wavelength-by-Wavelength temperature-independent thermal radiation utilizing an insulator-metal transition. *ACS Photonics*.

[39] Stolberg-Rohr T., Hawkins G. J. (2015). Spectral design of temperature-invariant narrow bandpass filters for the mid-infrared. *Opt. Express*.

[40] Rodrigues J., Bhatt G., Datta I. (2022). SiN-based waveguides with ultra-low thermo-optic effect. *Conference on Lasers and Electro-Optics, Technical Digest Series*.

[41] Kuznetsov A. I., Miroshnichenko A. E., Brongersma M. L., Kivshar Y. S., Luk’yanchuk B. (2016). Optically resonant dielectric nanostructures. *Science*.

[42] Yeshchenko O. A., Bondarchuk I. S., Gurin V. S., Dmitruk I. M., Kotko A. V. (2013). Temperature dependence of the surface plasmon resonance in gold nanoparticles. *Surf. Sci*..

[43] Ujihara K. (1972). Reflectivity of metals at high temperatures. *J. Appl. Phys*..

[44] Bouillard J. S. G., Dickson W., O’Connor D. P., Wurtz G. A., Zayats A. V. (2012). Low-temperature plasmonics of metallic nanostructures. *Nano Lett*..

[45] Collrnst R. J., Fan A. H. Y. (1954). Infrared lattice absorption bands in germanium, silicon, and diamond. *Phys. Rev.*.

[46] Orfanidis S. J., Monica T. (2008). *Electromagnetic Waves and Antennas*.

[47] Yu N., Capasso F. (2014). Flat optics with designer metasurfaces. *Nat. Mater.*.

[48] Singh D., Poplinger M., Twitto A. (2022). Chemical vapor deposition of spherical amorphous selenium Mie resonators for infrared meta-optics. *ACS Appl. Mater. Interfaces*.

[49] Zhang P., Guan J. (2011). Fabrication of multilayered microparticles by integrating layer-by-layer assembly and microcontact printing. *Small*.

[50] Zuev D. A., Makarov S. V., Mukhin I. S. (2016). Fabrication of hybrid nanostructures via nanoscale laser-induced reshaping for advanced light manipulation. *Adv. Mater.*.

[51] Mansha S., Moitra P., Xu X. (2022). High resolution multispectral spatial light modulators based on tunable fabry-perot nanocavities. *Light Sci. Appl.*.

[52] Shcherbakov M. R., Liu S., Zubyuk V. V. (2017). Ultrafast all-optical tuning of direct-gap semiconductor metasurfaces. *Nat. Commun.*.

[53] Decker M., Staude I., Falkner M. (2015). High-efficiency dielectric Huygens’ surfaces. *Adv. Opt. Mater.*.

[54] Iyer P. P., Butakov N. A., Schuller J. A. (2015). Reconfigurable semiconductor phased-array metasurfaces. *ACS Photonics*.

[55] Zhou T., Zhang J.-F., Hu B.-Q., Yang H.-G. (1994). Measurements of the thermo-optic coefficient of a barium fluoride single crystal. *Appl. Opt*.

[56] Malitson I. H. (1964). Refractive properties of barium fluoride*. *J. Opt. Soc. Am.*.

[57] Evlyukhin A. B., Reinhardt C., Seidel A., Luk’Yanchuk B. S., Chichkov B. N. (2010). Optical response features of Si-nanoparticle arrays. *Phys. Rev. B Condens. Matter Mater. Phys.*.

