# Supplementary material for: Temperature invariant metasurfaces
Source: Nanophotonics. 2023 Jun 22;12(16):3217–27. doi: 10.1515/nanoph-2023-0075 (PMC11501816; doi:10.1515/nanoph-2023-0075)
Supplement: Supplementary file 1 — Supplementary Material Details [file j_nanoph-2023-0075_suppl_001.pdf]

Supporting Information

Temperature Invariant Metasurfaces

Shany Zrihan Cohen[1,2], Danveer Singh[1,2], Sukanta Nandi[1,2] and Tomer Lewi[1,2]

[1]Faculty of Engineering, Bar-Ilan University, Ramat-Gan 5290002, Israel
[2]Institute for Nanotechnology and Advanced Materials, Bar-Ilan University Ramat-Gan 5290002, Israel
[*]Corresponding authors: tomer.lewi@biu.ac.il

1. The Thermo-Optic Effect

The thermo-optic (TO) effect describes the variation of the refractive index with temperature, and can be defined in the transparent regime as [1]:

$$(1)\ \ 2n\frac{dn}{dT} = (n_\infty^2 - 1)\left(-3\alpha R - \frac{1}{E_{eg}}\frac{dE_g}{dT}R^2\right)$$

where n, n_∞ and T are the refractive index, the high frequency refractive index and temperature respectively, α is the linear thermal expansion coefficient, and $R = \frac{\lambda^2}{\lambda^2 - \lambda_{ig}^2}$ where λ_{ig} is the wavelength corresponding to the temperature-invariant isentropic bandgap [1], and E_{eg} is the temperature-dependent excitonic bandgap.

As the relation in Eq 1. suggests - the TO coefficient encapsulates contributions from several physical mechanisms such as thermal expansion, excitonic and phonon excitations. For semiconductors and dielectrics, operating in the normal spectral regime (for $\lambda > \lambda_{ig}$ and therefore R>0) – the dominant contributor is the excitonic bandgap, typically by two orders of magnitude (α is typically ~10^{-6}/°K while dE_{eg}/dT ~10^{-4}eV/°K). For vast majority of semiconductors (Silicon included), the excitonic bandgap decreases with temperature $(\frac{dE_{Eg}}{dT} < 0)$ and since it is multiplied by another negative term (-1/ E_{eg}), it follows that the TO coefficient is usually positive. Interestingly, the lead chalcogenide family PbX (X=Te, Se, S) - exhibit an anomalous bandgap energy dispersion with temperature $dE_g/dT>0$ and hence also a similar behavior for the excitonic bandgap dispersion $dE_{eg}/dT>0$, i.e., the bandgap increases when temperature increases [2] and

hence the refractive index in lead chalcogenides decreases with temperature, in contrast to most materials. This anomalous negative TO coefficient in PbTe is the enabling component for the TO dispersion engineering presented in this work.

Throughout this work we used the wavelength and temperature dependent TO coefficients as detailed in ref [1], where dn_{Si}/dT = 2.5 [$10^{-4}/^{0}K$], dn_{PbTe}/dT = -13.5 [$10^{-4}/^{0}K$] are typical values in the transparent region. The chromatic dispersion of Si at room temperature (RT) was taken from Palik [3] and the dispersion relation of PbTe is given by [4,5] :

$$(2)\ n_{PbTe}(\lambda) = \sqrt{1 + \frac{30.586\lambda^2}{\lambda^2 - 2.0494} - 0.0034832\lambda^2}$$

When calculating thermal and TO effects, we assumed materials and components have reached equilibrium and did not consider any transient effects. We also did not consider any nonlinear effects. For PbTe, we used the TO coefficient at RT [1], since experimental measurements at other temperatures are missing.

2. Field Profiles

2.1 Multilayer core-shell Spherical Resonators

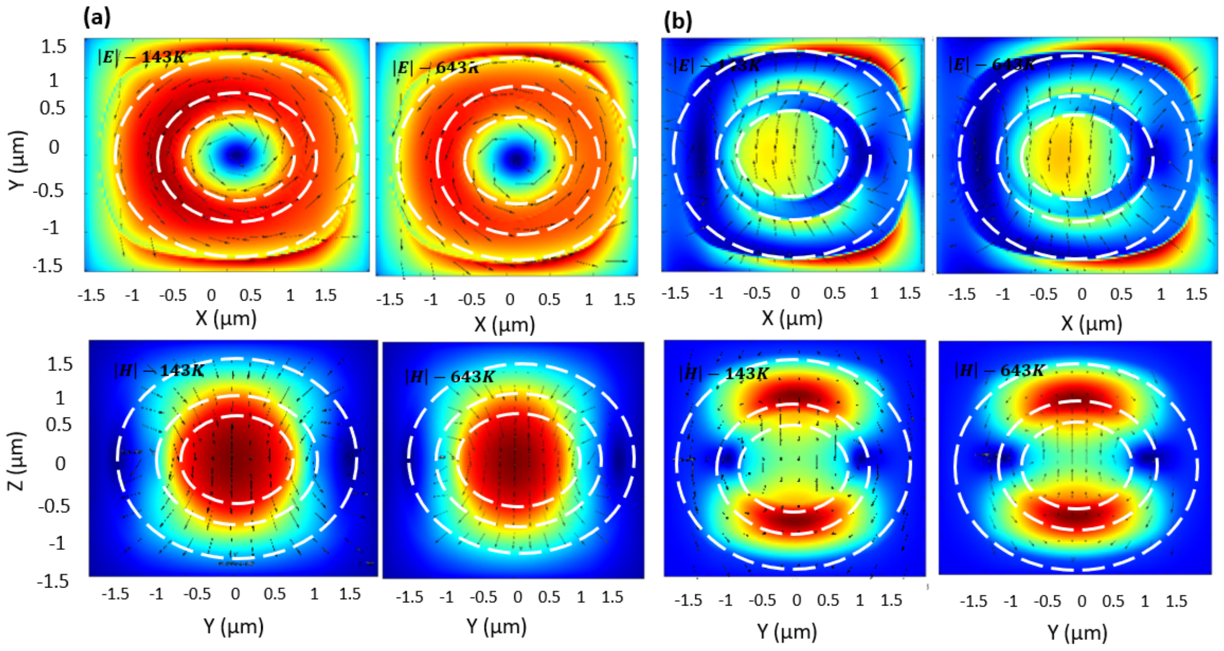

Figure S1. Electric and magnetic field profiles along the xy and yz planes for the first two fundamental resonances, calculated at the lowest (143K) and highest (643K) temperatures for the hybrid sphere presented in Figure 4 of main text. (a) Fundamental MD resonance at $\lambda \cong 9.25\mu m$ (b) ED resonance at $\lambda \cong 7.22\mu m$. The 3-layer hybrid sphere dimensions are r_{Si} =0.43μm, r_{PbTe} = 0.62μm and the outer Si radius is r_{Si} = 1.2μm.

The field distributions of the first two resonant modes of the multilayer hybrid spherical resonators, presented in the main text are shown in Fig S1. The first resonance at $\lambda \cong 9.25\mu m$ (Fig S1a) shows a clear distribution of a magnetic dipole (MD) mode. The second resonance at $\lambda \cong 7.22\mu m$ (Fig S1b) resembles an electric dipole (ED), however the field pattern is more complex compared to a single sphere, due to the additional shell layers. Interestingly, field profiles of both modes seem almost identical in the two extreme temperature values $143K\ and\ 643K$.

2.2 Multilayer hybrid Cubic and disk resonators

Figure S2 and S3 present the electric and magnetic field patterns of the cubic and disk structures at the fundamental MD resonance, at the lowest (143K) and highest (643K) temperatures.

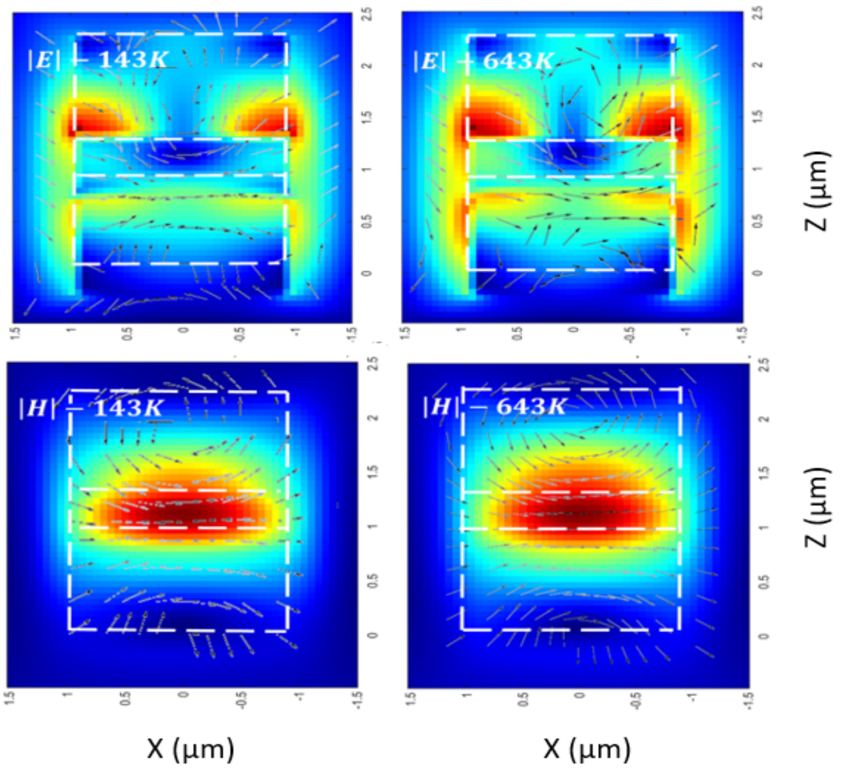

Figure S2 Electric and magnetic field patterns (xz plane) of the cubic resonator at the first resonance λ=5.86µm. the cubic geometry is: h_{Si} = 0.92µm, h_{PbTe} = 0.34µm, while the length and width at the x-y plane are 1µm each

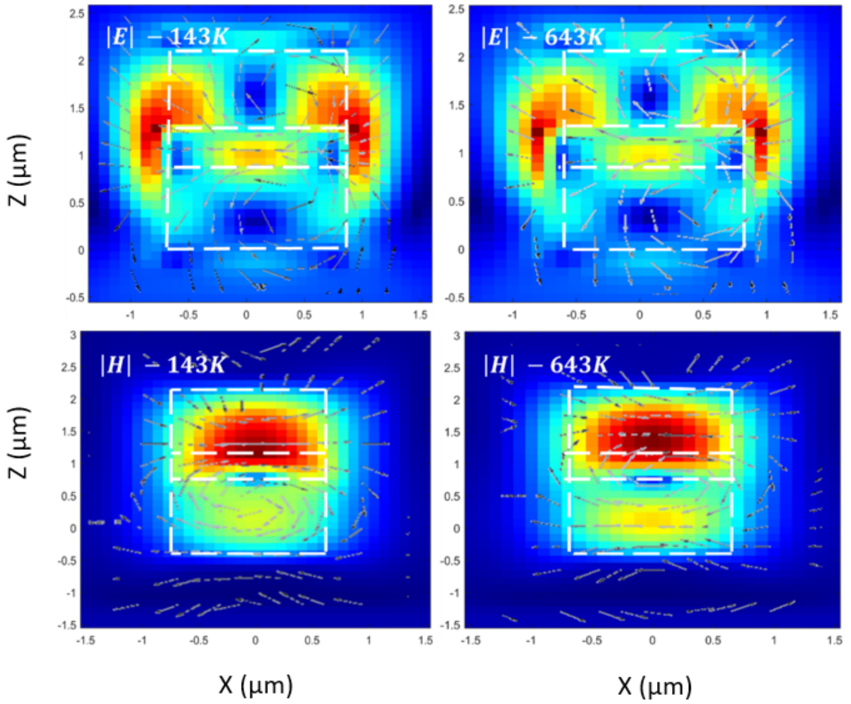

Figure S3 Electric and magnetic field patterns (xz plane) of the disk resonator at the second resonance λ=4.71µm. The disk geometry is h_{Si} = 0.96µm, h_{PbTe} = 0.24µm, r=0.5µm.

2.3 Hybrid disk metasurface

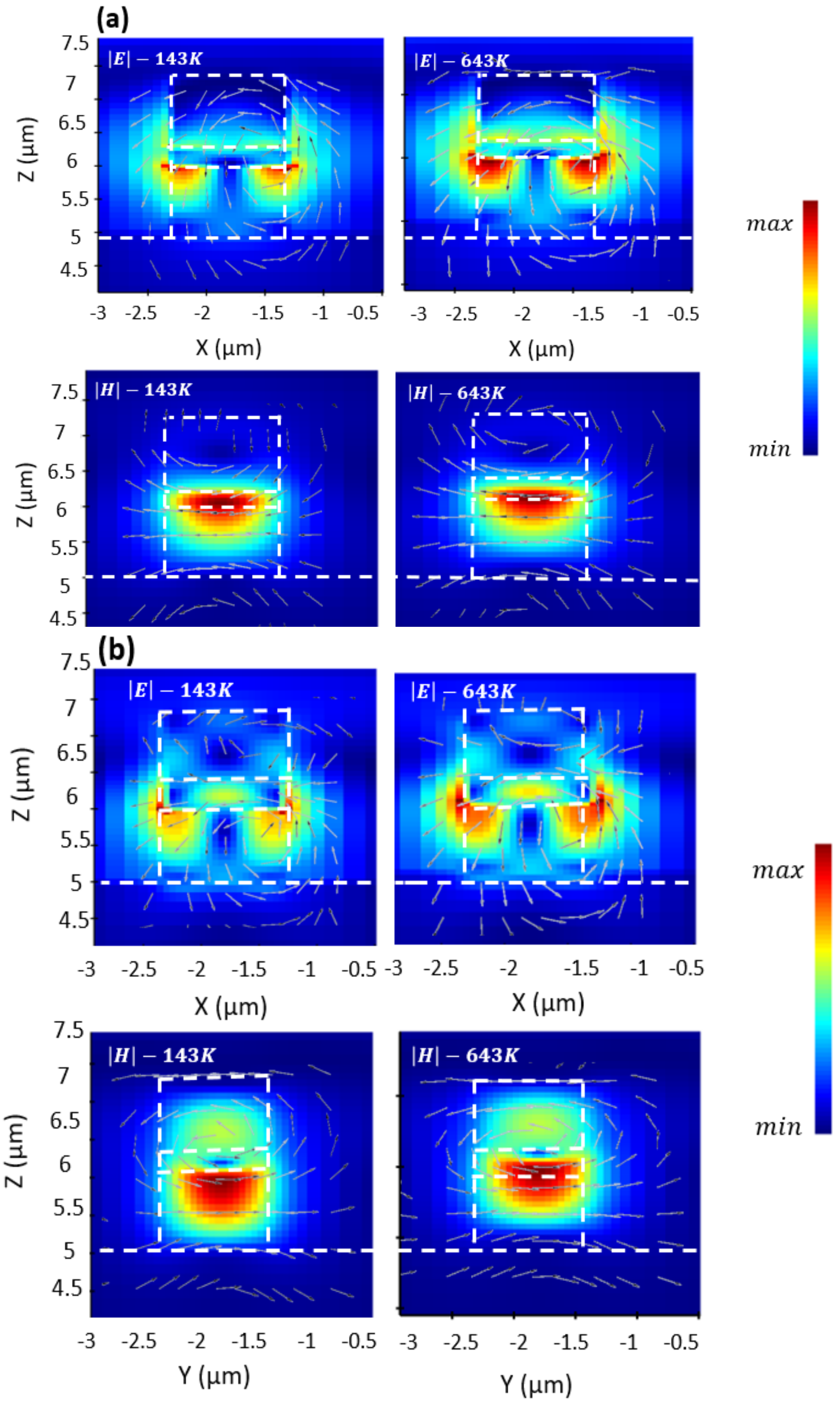

Figure S4: Electric and magnetic field patterns for the first two resonances of the hybrid unit cell disk metasurface in figure 8 of main text. The images compare the field profiles at the lowest and highest temperatures – (a) Fundamental resonance at λ≈5μm demonstrating MD pattern (b) second resonance at λ≈4.66μm demonstrating ED pattern. The hybrid disk resonator geometry is h_{Si} =1μm, h_{PbTe} = 0.25μm, r = 1μm and the periodicity is Λ 3.2μm

3. Spectra of uncompensated TO structures

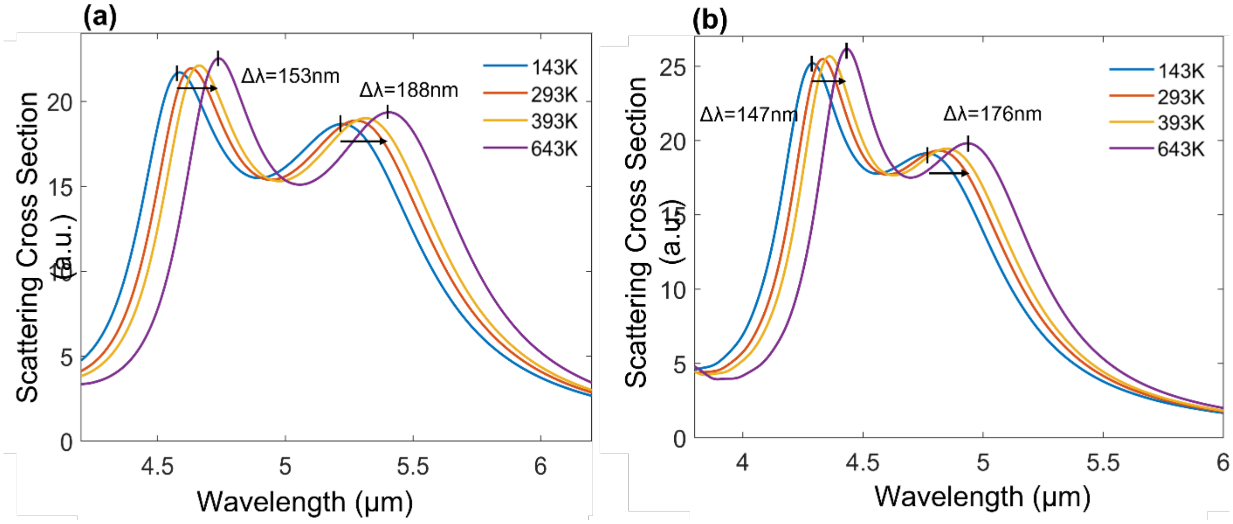

Figure S5: Spectra of uncompensated TO structures of uniform Si for (a) cubic and (b) disk geometries. The size (volume) of the resonators in both cases match to the size (volume) of the corresponding resonators in Figure 6 of main text. In both cases the spectra exhibit significant redshifts, manifesting undesirable temperature sensitive properties, as opposed to the temperature invariant hybrid structures presented in Figure 6 of the main text.

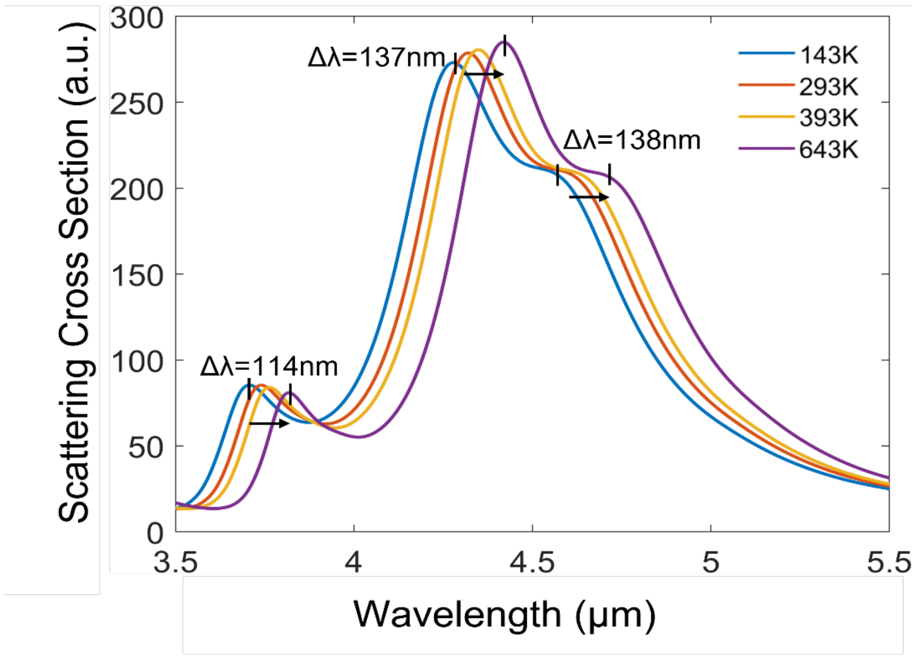

Figure S6: Spectra of uncompensated TO metasurface comprised of uniform Si disk resonators on a BaF_2 substrate. Each resonator unit-cell has the same size of the hybrid unit cell presented in the Figure 8 of main text. Compared to the temperature invariant hybrid disk metasurface presented in Figure 8 of the main text, the spectra of the Si metasurface exhibits significant resonance shifts. This resonance shifts will also cause significant changes to the phase.

4. Numerical Simulations

Finite difference time domain (FDTD) calculations were performed using the Lumerical Solutions FDTD solver, Version 8.25.2647. New materials with the required dispersion data were imported to Lumerical data set. The excitation source in all cases is a plane wave.

Simulations of Cross Section Scattering

These simulations were computed using the 'cross-section' analysis object. The simulation region consisted of each of the structures surrounded by air. A non-uniformal conformal mesh was used. A mesh size of at least 10x smaller than the minimum wavelength in the material was used with boundary conditions of perfectly matched layer.

Simulations of Reflection and Transmission Metasurfaces

These simulations were computed using the 'Grating S parameters' analysis object. The simulation region consisted of a full metasurface comprised of each structure (including substate, whenever applies) surrounded by air. PML boundary conditions were used in the radiation axis $\hat{z}$, while periodic symmetric and antisymmetric boundary conditions were used in the $\hat{x}, \hat{y}$ directions, respectively. The boundary conditions are selected with respect to the polarization direction and dictates the electric and magnetic field components which are zero at the plane of symmetry [6–8]

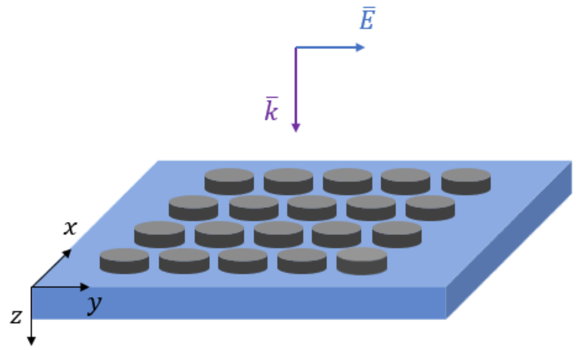

Figure S7: The simulated metasurface schematic

5. References

[1] Gorachand Ghosh, *Handbook of Thermo-Optic Coefficients of Optical Materials and Applications* (1997).

[2] T. Lewi, H. A. Evans, N. A. Butakov, and J. A. Schuller, *Ultrawide Thermo-Optic Tuning of PbTe Meta-Atoms*, Nano Lett **17**, 3940 (2017).

[3] E. D. Palik, B. San, D. New, Y. London, and S. T. Toronto, Handbook of Optical Constants of Solids II ACADEMIC PRESS, INC. Harcourt Brace Jovanovich, Publishers, n.d.

[4] https://refractiveindex.info/?shelf=main&book=PbTe&page=Weiting-300K

[5] F. Weiting and Y. Yixun, TEMPERATURE EFFECTS ON THE REFRACTIVE INDEX OF LEAD TELLURIDE AND ZINC SELENIDE, 1990.

[6] R. C. Thompson, *Optical Waves in Layered Media*, J Mod Opt **37**, 147 (1990).

[7] Z. Szabó, G. H. Park, R. Hedge, and E. P. Li, *A Unique Extraction of Metamaterial Parameters Based on Kramers-Kronig Relationship*, IEEE Trans Microw Theory Tech **58**, 2646 (2010).

[8] D. R. Smith, D. C. Vier, T. Koschny, and C. M. Soukoulis, *Electromagnetic Parameter Retrieval from Inhomogeneous Metamaterials*, Phys Rev E Stat Nonlin Soft Matter Phys **71**, (2005).

[9] T. Lewi, N. A. Butakov, and J. A. Schuller, *Thermal Tuning Capabilities of Semiconductor Metasurface Resonators*, Nanophotonics **8**, 331 (2018).
